# Supplementary material for: Long-Term Results in Minimally Invasive Non-Resectional Mitral Valve Repair for Barlow Mitral Valve Disease
Source: J Clin Med. 2025 Feb 5;14(3):1005. doi: 10.3390/jcm14031005 (PMC11818547; doi:10.3390/jcm14031005)

Supplemental Materials

Tables

Table 1 Baseline echocardiographic and intraoperative characteristics

	Total (N=98)
**Echocardiographic characteristics**	
LVEF	60 (8.8)
**Mitral valve regurgitation grade**	
Moderate	2 (2.0%)
Severe	69 (97.9%)
**Operative characteristics**	
Emergency operation	1 (1.0%)
Cardiopulmonary bypass time (min)	147 (45)
Cross clamp time, (min)	91 (29)
**Neo-Chordae to the target leaflet**	
Isolated anterior leaflet	6 (6.1%)
Isolated posterior leaflet	44 (44.8%)
Anterior and posterior leaflet	7 (7.1%)

LVEF; left ventricular ejection fraction
Values are n (%) for categorical variables or mean and standard deviation for continuous variables

Figure legends

Figure 1:

Kaplan Meier plot for freedom from adverse events; myocardial infarction A), stroke B) of congestive heart failure C) and in patients following non minimal invasive non-resectional mitral valve repair for Barlow disease

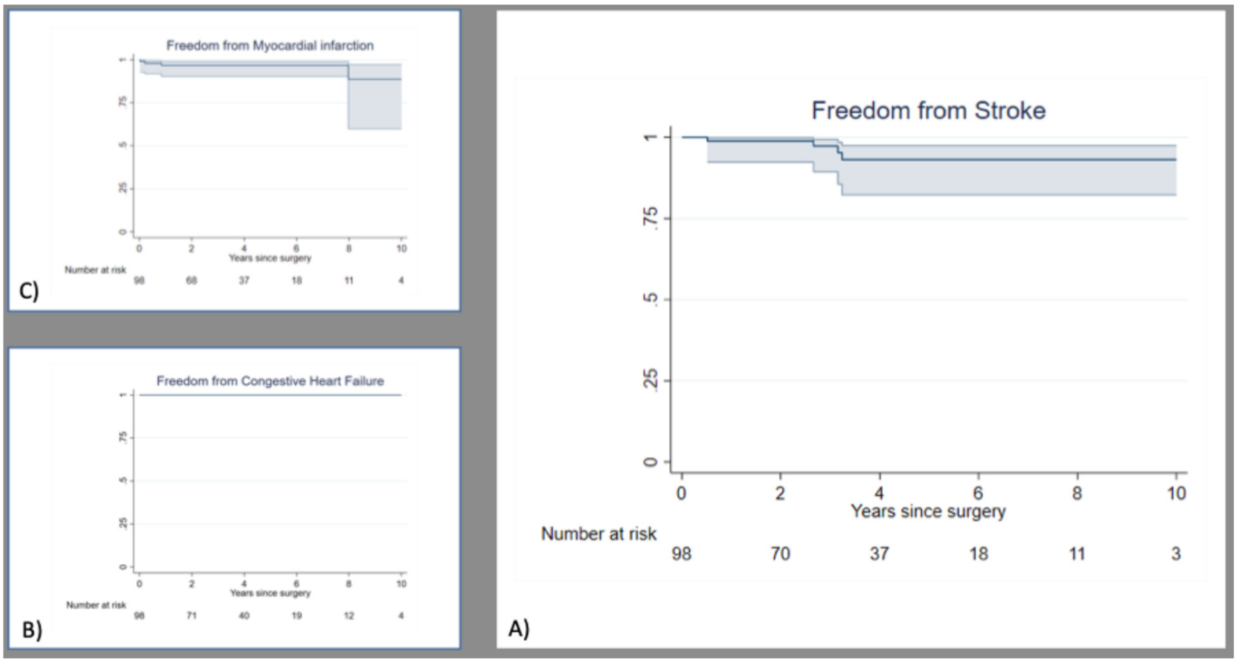

Supplement: Supplementary file 1 [file jcm-14-01005-s001.zip › jcm-3410251-supplementary.pdf]
